# Optimized Ion-Sensitive Hydrogels Based on Gellan Gum and Arabinogalactan for the Treatment of Dry Eye Disease

**DOI:** 10.3390/gels11100787

**Published:** 2025-10-01

**Authors:** Valentina Paganini, Silvia Tampucci, Sofia Gisella Brignone, Mariacristina Di Gangi, Daniela Monti, Susi Burgalassi, Patrizia Chetoni

**Affiliations:** 1 Department of Chemistry and Industrial Chemistry, University of Pisa, I-56126 Pisa, Italy; valentina.paganini@dcci.unipi.it; 2 Department of Pharmacy, University of Pisa, I-56126 Pisa, Italy; silvia.tampucci@unipi.it (S.T.); sofiagisella.brignone@farm.unipi.it (S.G.B.); mariacristina.digangi@phd.unipi.it (M.D.G.); daniela.monti@unipi.it (D.M.); patrizia.chetoni@unipi.it (P.C.); 3 Inter-University Center for the Promotion of the Rs Principles in Teaching & Research (CentroR), I-56126 Pisa, Italy

**Keywords:** arabinogalactan, gellan gum, hydrogel, dry eye disease, wettability, rheology, mucoadhesion, precorneal retention

## Abstract

Dry eye disease (DED) is a multifactorial condition characterized by insufficient tear film stability and ocular discomfort. Conventional artificial tears offer limited efficacy due to short precorneal residence time. This study aimed to develop and optimize ion-sensitive in situ gelling formulations based on low-acyl gellan gum (GG) and arabinogalactan (AG) to enhance retention and therapeutic efficacy in DED. Various buffer systems were screened to identify optimal gelation conditions upon interaction with artificial tear fluid (ATF). Formulations were characterized by pH, osmolality, wettability, thermal behavior, viscosity, and viscoelastic properties. A Design of Experiments (DoE) approach was employed to understand the influence of GG and AG concentrations on rheological behavior. The selected formulation, GG(0.1%)/AG(0.2%), demonstrated a significant viscosity increase upon ATF dilution, suitable viscoelastic properties, enhanced mucoadhesion compared to hyaluronic acid, improved ferning patterns, no cytotoxic effects, and stability over time. In vivo studies in rabbits confirmed prolonged precorneal retention of the fluorescently labeled formulation. These results suggest that the GG/AG-based hydrogel is a promising strategy for improving the performance of artificial tears in DED treatment.

## 1. Introduction

The management of dry eye disease (DED) presents significant challenges for healthcare professionals due to the complex interplay of factors contributing to this chronic condition. Topical ophthalmic therapy with artificial tears remains the first-line treatment; however, the bioavailability of these formulations is severely limited by the eye’s protective mechanisms. It is estimated that only 1–2% of the administered dose reaches the anterior segment of the eye [1]. Considering the limited efficacy of conventional eye drops, considerable attention has been directed toward advanced drug delivery systems capable of enhancing drug residence time on the ocular surface.

Among these, in situ gelling systems have been shown to be particularly promising as they can form hydrogels able to absorb water while maintaining structural integrity. This allows them to resist removal by tearing and blinking, thereby prolonging their residence time in the precorneal area [2]. Ion-sensitive hydrogels are especially relevant in ophthalmology as they respond to the ionic changes in tear fluid, improving ocular surface adhesion and drug retention [3].

Arabinogalactan (AG) is a water-soluble polysaccharide obtained mainly from the bark of plants of the Larix genus, consisting of arabinose and galactose in a ratio of 1:6, along with small amounts of glucuronic acid [4,5]. Recently, AG has attracted the attention of researchers, and several studies have been published regarding its combination with hyaluronic acid for the development of ophthalmic products intended for DED management [6,7,8]. Although AG has demonstrated biocompatibility, therapeutic utility in DED, and the ability to support corneal re-epithelialization, it is characterized by low viscosity and Newtonian flow behavior, even at high concentrations [9,10].

The present study therefore focused on formulating a system with improved retention performance on the ocular surface in order to maximize the therapeutic potential of AG.

Specifically, we aimed to develop ion-sensitive hydrogels by combining low-acyl gellan gum (GG) with AG to obtain an in situ gelling system that would extend the precorneal residence time and improve patient compliance by reducing the frequency of eye drop administration. Gellan gum, an anionic polysaccharide derived from Sphingomonas elodea, exhibits versatile gel-forming properties depending on its degree of acylation. The low-acyl variant produces transparent and less elastic gels, making it well suited for in situ gelling formulation. Its ability to undergo gelation in the presence of physiological ions makes it a promising candidate for ophthalmic formulations [11,12].

The first step of the research involved optimizing a GG-based formulation to combine with AG for use as an ocular in situ gelling system. The formulation performance was evaluated based on key parameters, such as sol–gel transition upon contact with artificial tear fluid. A preliminary screening was conducted using different buffer solutions to achieve optimal gelation when interacting with artificial tear fluid, analyzing factors such as pH, osmolality, and viscosity.

Subsequently, the preparation method for the formulations was refined, focusing on GG at 0.1% *w*/*w* alone and in combination with AG at concentrations of 0.2% and 0.3% *w*/*w*. Rheological and wetting analyses were instrumental in guiding the optimization process, ensuring both reproducibility and performance. A Design of Experiments (DoE) approach was adopted to evaluate the viscosity and viscoelastic properties of formulations containing GG (0.05%, 0.1%, and 0.2% *w*/*w*) with or without AG (0.2% and 0.3% *w*/*w*).

The selected in situ gelling formulation was further characterized by assessing mucoadhesive properties and performing Ferning tests to determine whether the polymeric dispersion, optimized in both composition and concentration, could mimic the behavior of natural tears in the presence of salts. Finally, the retention time of the formulation in the precorneal area was evaluated in rabbits as a key parameter to potentially enhance AG therapeutic effects on the corneal surface.

## 2. Results and Discussion

### 2.1. Pre-Formulative Study

#### 2.1.1. Optimization and Selection of the Buffer Solution

The preliminary phase of the study focused on optimizing the salt composition of the buffer solution (BS) to achieve a significant increase in viscosity upon dilution with artificial tear fluid (ATF). Indeed, the goal was to identify the optimal concentrations of monovalent and divalent cations in BS to obtain a formulation that already had a certain degree of viscosity at the time of administration but was still droppable, and which ensured complete gelation once in contact with the ions found in the tear fluid, while producing formulations that are both isohydric and isotonic with tears. By inducing gelation of the formulation after administration, it could be possible to improve its performance in the precorneal area.

All BS were composed of a constant amount of Na_2_HPO_4_ and citric acid (basic components of the buffer system), as well as NaCl, KCl and mannitol, but varied in their concentrations of CaCl_2_ (anhydrous). NaCl, KCl, and CaCl_2_ were chosen because they were present in the natural tear. The compositions and ionic concentrations of the five tested BS are detailed in Table 1 and reported in mM units. During the optimization process, the concentration of CaCl_2_ was increased from 0.76 mM to 2.00 mM, reaching a concentration recommended by Kelcogel for the development of low-acyl gellan gum (GG) gels that are sufficiently fluid for ophthalmic application (range of 2–5 mM). Additionally, the behavior of the formulation in the absence of CaCl_2_ was evaluated to assess the specific impact of Ca^2+^ ion on gel formation.

The characterization of the formulations presented in Table 2 reveals information on the impact of buffer solutions on the rheological properties of GG-based dispersions. The osmolality and pH of ophthalmic formulations should fall within the physiological range to avoid adverse effects on the eye. All formulations exhibited acceptable pH, ranging from 6.45 to 6.97, and osmolality values ranging from 292 to 297 mOsm/kg, very similar to those of natural lacrimal fluid. Although it is very often reported in the literature that the osmolarity of tear fluid is around 302 mOsmol/kg, some authors have demonstrated that physiologically, the osmolarity of the tear film is approximately 289 ± 21 mOsm/L and follows a circadian rhythm; therefore, values oscillating between 280 and 310 can be considered physiological [13,14]. It should be noted that the eye has a great compensatory capacity for pH and osmolarity due to high tear turnover. For the treatment of DED, it is commonly accepted that hypotonic artificial tear formulations are better than isotonic ones [15,16,17] as they reduce the tear hyperosmolarity, one of the factors playing a key role in the pathogenesis of DED [18]. However, precisely because of the rapid clearance of the tears and eye drops, the benefit in the administration of hypotonic tear substitutes has been shown to be minimal since the osmolarity returns to pre-administration values in a few seconds [19].

The formulations were subjected to viscosity measurements taken before and after ATF dilution at a 30:7 ratio. This ratio was chosen as optimal for mimicking physiological conditions. Specifically, this ratio reflects the typical volume of an eye drop (approximately 30 µL) relative to the volume of tear fluid in the conjunctival sac (about 7 µL), during the blinking process, as described by Kotreka et al. [20].

The results of the viscosity measurements highlighted a pseudoplastic, non-Newtonian behavior of GG dispersions, where viscosity drops markedly under shear due to chain alignment and partial disentanglement. The buffer composition and ion concentration have a remarkable impact on viscosity values. GG(0.1)/BS3 formulation, containing only monovalent ions (40.48 mM), exhibited low viscosity, i.e., about 2.18 mPa·s, which increased to 8.97 mPa·s upon contact with ATF. As expected, however, 0.10% GG dispersions prepared in BS containing CaCl_2_ exhibited higher viscosity values, although no differences were evident between the two CaCl_2_ concentrations used in BS1 and BS2; the viscosity values were 28.62 ± 0.66 and 28.54 ± 2.11 mPa·s for formulations with 0.76 and 2.00 mM of Ca^2+^, respectively. The formulation GG(0.1)/BS1, with the lower concentration of Ca^2+^, demonstrated a noticeable increase in viscosity after dilution with ATF, rising from 28.62 ± 0.66 to 31.27 ± 0.64 mPa·s. This suggests that while the formulation is stable with lower Ca^2+^ concentrations, its viscosity is further enhanced by the presence of additional ions in the ATF. Ionic strength, through salt or buffer cations, screens electrostatic repulsions, promotes coil–helix transitions of the GG chain, and strengthens transient junctions, increasing low-shear viscosity in gellan systems [21]. Conversely, the formulation GG(0.1)/BS2, with a higher concentration of Ca^2+^, showed a slight decrease (not statistically significant) in viscosity after dilution with ATF, i.e., from 28.54 ± 2.11 to 26.48 ± 1.09 mPa·s. The total ionic concentration of gelling systems based on low-acyl gellan gum is known to be a critical factor for gelation: increasing the Ca^2+^ concentration causes gelation up to a critical point, beyond which a progressive reduction in viscosity occurs. When the level of counter-ions is sufficient to induce network formation, any further addition may lead to destabilization of the system [22].

Our results highlight that a correct ratio between GG and cations can be crucial for achieving a significant increase in viscosity. The preliminary evaluation of buffer solutions led to the selection of BS1 as it had a certain degree of viscosity after preparation and was effective in significantly increasing viscosity upon dilution, with ATF demonstrating that it strikes a favorable balance in ion composition, making it an appropriate choice for an in situ gelling formulation intended for ophthalmic application.

#### 2.1.2. Characterization of GG-Based Formulations and Their Mixtures with AG

This study aimed to characterize formulations based on GG at concentrations between 0.05 and 0.2%, also with the addition of arabinogalactan (AG) at concentrations of 0.2 or 0.3% *w*/*w*. The formulations were prepared using the selected buffer, BS1, where the appropriate amount of GG was hydrated by heating (80 °C on a water bath) before adding the eventual amount of AG. Afterward, the solution was allowed to cool to room temperature, and benzalkonium chloride (BAK, 0.005% *w*/*w*) as a preservative, EDTA (0.050% *w*/*w*) as a stabilizer, and purified water to reach the desired weight were added.

The preparation method involved an appropriate heating step to promote the formation of the characteristic three-dimensional polymeric network, enabling the transformation into a viscous gelling dispersion. The final composition of the formulations under investigation is reported in Table 3, while Table 4 details the characterization parameters, including pH, osmolality, wettability, and viscosity before and after dilution with ATF.

The pH and osmolality values of the formulations remained within the acceptable ranges for ophthalmic use.

Wettability is a critical parameter for formulations intended for topical ophthalmic application, as enhanced wettability indicates a stronger affinity between the formulation and the ocular surface, allowing for better spreading and adhesion on the cornea. The wettability of the formulations was determined by measuring the contact angle, and all tested formulations exhibited values below 90°, ranging from 48.50° to 57.60°. It should be noted that these measurements were carried out on a substrate that was less hydrophilic than the tear-covered ocular surface, and that the formulations already exhibited a certain degree of viscosity even prior to dilution with ATF. In fact, polymer dispersions with non-Newtonian rheological behavior limit the distribution of the product on a surface [23], an effect known as viscous dissipation [24]; therefore, all the tested formulations can be considered to have good wettability.

Despite the increase in viscosity following dilution with ATF, the contact angle values remained largely stable. In some cases, a slight tendency towards higher values was observed; however, none exceeded 90°, thus maintaining the wettability of the formulations.

Rheological characterization of the prepared formulations was also conducted before and after dilution with ATF in a 30:7 volume ratio. For the entire series of formulations, the rheological behavior was found to be pseudoplastic. Pseudoplastic behavior is advantageous for ophthalmic formulations as it allows for ease of instillation and spread on the ocular surface during the blinking process.

This behavior mimics that of the natural tear film, which decreases its viscosity as the shear rate increases during blinking movements to avoid damage to corneal epithelial surface; however, during the interblink phase, it assumes a higher viscosity to resist drainage and break-up [25,26].

The viscosity of the formulations is strictly linked to GG concentration, while the addition of AG, at the concentration used, does not seem to exert influence on this parameter. The results obtained show that the average increase in viscosity values after mixing with ATF (in Table 4 as “Increase Factor, mean”) is directly proportional to the concentration of GG present in the formulation (R value of linear regression = 0.9985) regardless of the AG concentration added. Dilution with ATF always leads to an increase in the viscosity of the formulation, confirming the ability of the GG dispersion to gel in the presence of tear fluid, even after the addition of AG and excipients.

The combination of AG and GG appears to produce gelling systems with greater stability in final viscosity than those prepared by combining AG with HA. Indeed, Di Mola’s research group found a progressive decrease in viscosity as the AG concentration in the formulation increased for all shear rates tested [7].

Numerous studies have investigated the optimal viscosity range for ophthalmic formulations, indicating that 15–30 mPa·s may represent the upper limit of acceptable viscosity [27]. The use of highly viscous artificial tears has been associated with ocular discomfort, which may ultimately compromise patient compliance [28].

It is well established that tear fluid possesses not only viscous but also elastic properties [29,30]. Therefore, the viscoelastic behavior of the formulations was investigated using stress sweep (to identify the linear viscoelastic region) and frequency sweep tests for a comprehensive rheological characterization. The frequency sweep analysis provided detailed insights into the viscoelastic properties of the formulations, including the elastic modulus (G′), viscous modulus (G″), and phase angle (tan δ).

**Figure 1:**
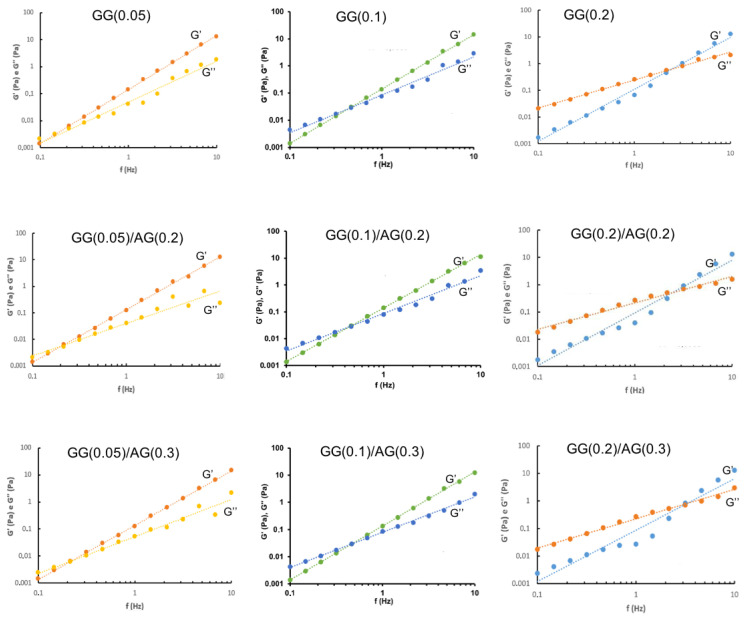
Frequency sweep of the formulations under study before ATF mixing.

**Figure 2:**
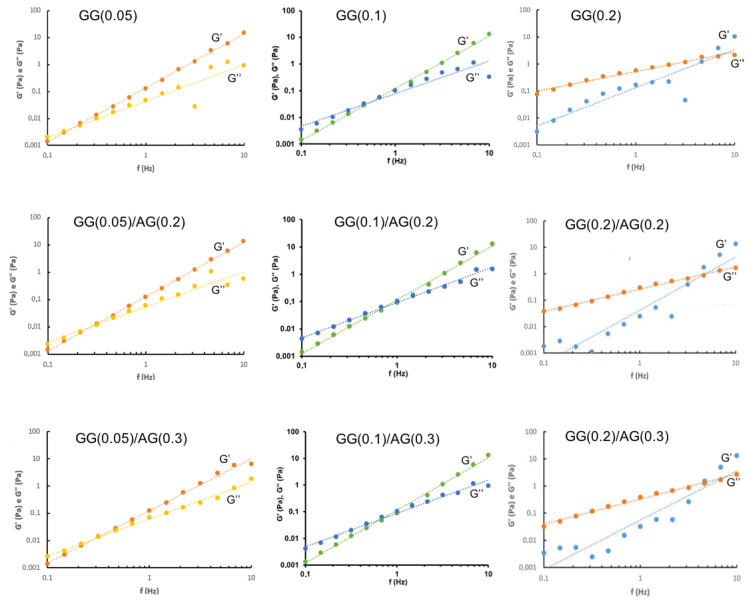
Frequency sweep of the formulations under study after ATF mixing.

Figure 1 and Figure 2 show the graphs of the experimental points exhibiting the trend of the elastic and viscous moduli of the studied formulations as the oscillation frequency varies, respectively, before and after dilution with ATF. Also in this case, it should be noted that the rheological profiles are a function of GG concentration in the formulation and that they are only influenced a little by the addition of AG.

The formulations at the lowest GG concentration (0.05%) show elastic behavior, even at low frequencies (<0.5 Hz), demonstrating that these materials are very resistant to deformation when a force is applied. As the GG concentration increases, the formulations show a more viscous character, and in all the GG(0.2)-based formulations, the viscous behavior prevails over the elastic one—a typical trend of a material with a rigid consistency at rest, which is easily deformable by applying a force. Frequency sweep profiles in which the viscous modulus dominates at low frequency values, while at higher frequencies the elastic modulus prevails represent the viscoelastic behavior of fluid (liquid-like) hydrogels [30].

The crossover point, where the elastic modulus exceeds the viscous one, moves to higher frequency values after mixing with ATF, especially in the case of the GG(0.2) series formulations where it occurs at values around 5–6 Hz. This important elastic component, which remains intact even after dilution with ATF, keeps the formulation in its gel state, even at blink frequencies. While the high elastic component is essential for the stability of the gel network, it is not beneficial for the eye as it can cause discomfort in the patient during blinking [31].

#### 2.1.3. Design of Experiment (DoE) Optimization Study

The data obtained from the rheological analysis on the formulations under study were used to compute the response surface in order to understand how the two independent variables (X_1_ = AG% *w*/*w* and X_2_ = GG% *w*/*w*) influenced the rheological behavior of the formulation. The response surfaces of the two independent variables were calculated before and after mixing with artificial tear fluid for three dependent variables: viscosity (mPa·s) measured at 2.5 s^−1^, elastic modulus (Pa), and viscous modulus (Pa). In this optimization study, the wettability factor was not included as a variable as it had proven not to be discriminating between the various formulations.

The data used for the calculation are summarized in Table 5, where the viscoelastic parameters are calculated at 1 Hz using interpolation equations from the frequency sweep graphs.

**Figure 3:**
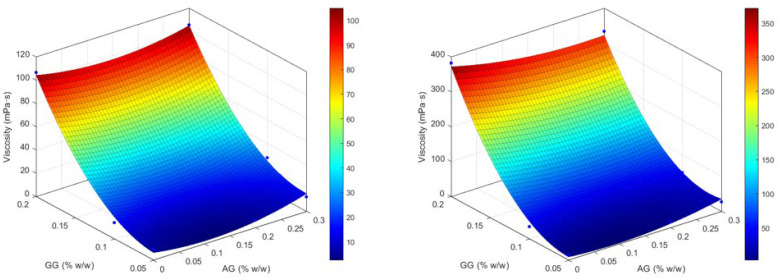
Response surfaces for experimental values of the viscosity of the formulations before (on the **left**) and after (on the **right**) dilution with ATF.

The analysis of the response surface in the first independent variable (Figure 3) clearly confirms how the variation in viscosity, both before and after mixing with ATF, depends exclusively on the amount of GG used, while it does not seem sensitive to the amount of AG; the higher GG, the higher viscosity is reached. Statistical modeling supports the following: the quadratic term of GG is highly significant, both without ATF (*p* = 0.0037) and with ATF (*p* = 0.0081), while AG-related and interaction terms remain non-significant. The model fits are excellent (R^2^ ≈ 0.9971 and 0.9947, respectively).

This is supported by the regression model, which shows a highly significant quadratic effect of GG (β_4_, *p* = 0.0037) and a significant linear effect (β_1_, *p* = 0.0383), whereas AG’s linear and quadratic terms (β_2_, β_5_) and the GG × AG interaction (β_3_) were not significant (all *p* > 0.2). Goodness of fit is excellent (R^2^ = 0.9971, F(5,3) = 203.2, *p* = 0.0005), although multicollinearity is high (VIF for GG~50.8, for GG^2^~49.0), especially among GG terms, but the GG quadratic component remains robust despite this.

**Figure 4:**
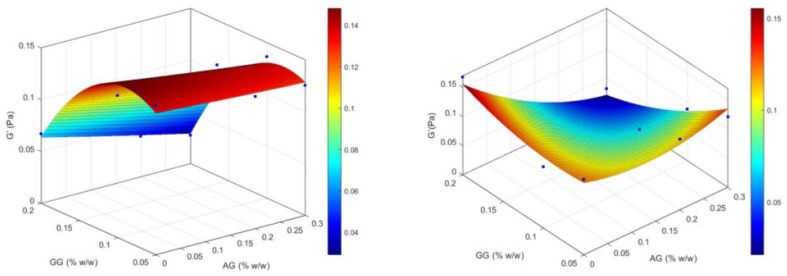
Response surfaces for experimental values of the elastic modulus of the formulations before (on the **left**) and after (on the **right**) dilution with ATF.

Conversely, the elastic modulus (Figure 4) tends to be higher for low concentrations of GG and is little affected by the amount of AG. In the tested series, after mixing with ATF, the highest G′ values are obtained for the formulations based on GG only, and the maximum G′ value is achieved at the maximum concentration of GG. The association with AG generally determines a reduction in the elastic modulus which is more marked after mixing with ATF and with increasing GG concentration, going from 0.121 Pa of GG(0.05)/AG(0.2) and GG(0.05)/AG(0.3) to 0.092 and 0.088 Pa, and finally to 0.025 and 0.032 Pa for GG(0.1)/AG(0.2), GG(0.1)/AG(0.3), GG(0.2)/AG(0.2) and GG(0.2)/AG(0.3), respectively. Before ATF, both linear (GG) and quadratic (GG^2^) terms for GG are significant predictors of G′; after ATF, only the GG × AG interaction is significant for G′ (*p* = 0.0441), indicating that the combined polymer concentrations influence elasticity under tear-fluid conditions.

**Figure 5:**
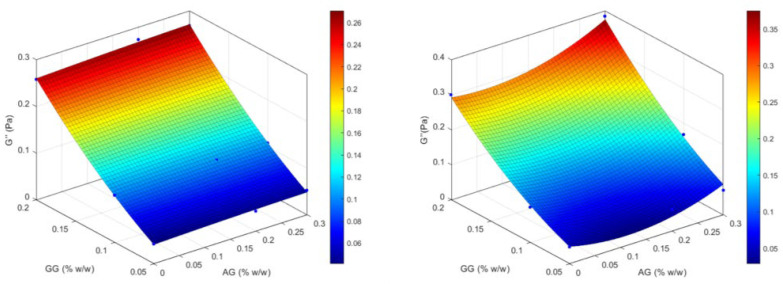
Response surfaces for experimental values of the viscous modulus of the formulations before (on the **left**) and after (on the **right**) dilution with ATF.

The same dependence on GG concentration is found in the response surfaces of the viscous modulus (G″), presented in Figure 5, before dilution with ATF, with values approximately 6-fold higher in the formulations of the GG(0.2) series, regardless of the amount of AG added. Although a significant increase in viscosity was observed following dilution with ATF, G″ values do not appear to be substantially affected by this mixing, which show only a weak tendency to increase. Statistical modeling confirms that for G″, GG linearly and quadratically contributes significantly without ATF (*p* = 0.0307 and *p* = 0.0290), whereas post-ATF, none of the GG, AG, or interaction terms are significant despite a strong global fit (R^2^ ≈ 0.9901, *p* = 0.0033).

In GG(0.1)/AG(0.2) mixtures, statistical modeling shows that GG’s quadratic effects dominate viscosity and G″ both with and without ATF, while in the case of G′ with ATF, the GG × AG interaction becomes significant. This suggests that increased ionic strength in ATF modifies chain dynamics: GG concentration still controls elasticity, but AG presence and, above all, buffer ions jointly influence elastic behavior.

From the results of the formulation study, GG(0.1)/AG(0.2) was identified as the optimal formulation among those containing the active ingredient, AG, as it demonstrated a significant increase in viscosity values after mixing with ATF (from 17.83 ± 1.96 to 41.66 ± 8.12 mPa·s), appreciable elastic properties which did not significantly decrease after dilution with ATF (from 0.141 to 0.092 Pa), and a substantially stable viscous modulus (0.106 Pa). These characteristics suggest that after administration, the formulation is still able to gel thanks to the presence of an optimal concentration of GG, and it maintains a certain degree of elasticity and a stable value of the viscous modulus, which does not lead to the assumption of discomforting effects on the patient, such as blurred vision or the accumulation of the formulation on the palpebral fissure and on the eyelashes.

#### 2.1.4. Interactions and Stability of the Selected Formulation

**Figure 6:**
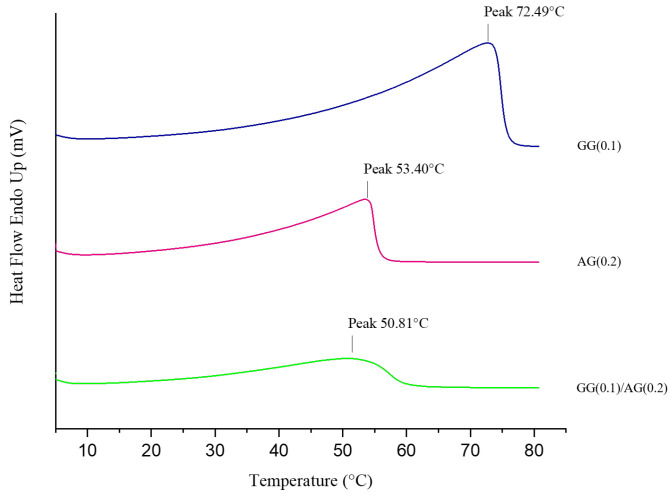
Thermal behavior of the selected formulation compared to the single components.

To determine any structural changes in the polymers GG and AG when mixed, Differential Scanning Calorimetry (DSC) analysis was carried out on three dispersion samples: GG(0.1), AG(0.2), and the DoE-selected formulation GG(0.1)/AG(0.2). The thermograms are shown in Figure 6. In the thermogram of the GG(0.1), an endothermic peak is observed at a temperature of 72.49 °C; for AG(0.2), the endothermic peak was observed at a temperature of 53.40 °C; and in the case of GG(0.1)/AG(0.2) formulation, the peak appears at a temperature of 50.81 °C.

These endothermic transitions are characteristic of polymeric systems; while the individual polymer dispersions exhibit distinct melting peaks at notably different temperatures, the mixture of GG and AG produces a less defined peak. This variation from the dispersions of the individual polymers can be attributed to the interactions that occur when the two polymers are combined. Specifically, when a semicrystalline polymer is mixed with an amorphous one, the crystalline structure of the semicrystalline polymer can be disrupted, resulting in a less defined melting peak compared to each polymer alone [32]. In our formulation, gellan gum, in its low-acetyl form, displays crystalline properties due to its double-helical structure stabilized by cations like potassium or calcium, which are present in the vehicle used for formulation preparation. This crystalline behavior stems from its organized polysaccharide chains, which are essential for its gelation capability [12]. On the other hand, arabinogalactan is predominantly amorphous because of its highly branched and flexible structure, which prevents the formation of ordered or crystalline arrangements [4].

That the two individual polysaccharides interact with each other is also evidenced by the results obtained by Dynamic Light Scattering (DLS) listed in Table 6. In fact, a statistically significant increase in hydrodynamic dimensions was found between those of the individual components and those obtained after their mixing in the final formulation, indicating a molecular interaction between the polysaccharides.

The data listed in Table 6 also show the stability of the final formulation to the filtration process, used as a sterilization method, and to storage. In fact, no statistically significant differences (one-way ANOVA) were highlighted either in the hydrodynamic size values or in the viscosity values between the GG(0.1)/AG(0.2) formulation before and after filtration and after its storage at room temperature for 9 months.

The stability results for the formulation are encouraging, as nine months is already a fairly long shelf life, although a longer shelf life is expected for an ocular formulation. Moreover, a possible rearrangement of the polymer interactions should lead to the measurement of populations into the dispersion with larger hydrodynamic dimensions or variations in viscosity values, which should already be evident after nine months.

### 2.2. Biopharmaceutical Evaluation of the Selected Formulation

#### 2.2.1. Ferning Test

The ferning test is conducted to assess whether the polymer dispersions can form crystalline structures like ferns observed in tear fluid after drying. Upon drying, tear fluid forms fern-like crystalline patterns, and this characteristic is also evaluated to diagnose forms of DED. Filippello and colleagues, in 1995, suggested applying the ferning test to polymer-based tear substitutes, assuming that those products that crystallized into fern-like structures, like tears, had a muco-mimetic characteristic useful in artificial tears [33]. The ferning are classified into four types based on the density and branching of the ferns: types I and II indicate a healthy tear film, while types III and IV suggest mucin degradation [34].

AG has been previously reported to form ferns, as demonstrated by Burgalassi et al. [9], who showed this ability at a concentration of 2.5%. However, in this context, it was necessary to confirm whether AG could still form ferns at a much lower concentration (0.2%) and to determine if this capacity was retained in the GG(0.1) in situ gelling dispersion.

**Figure 7:**
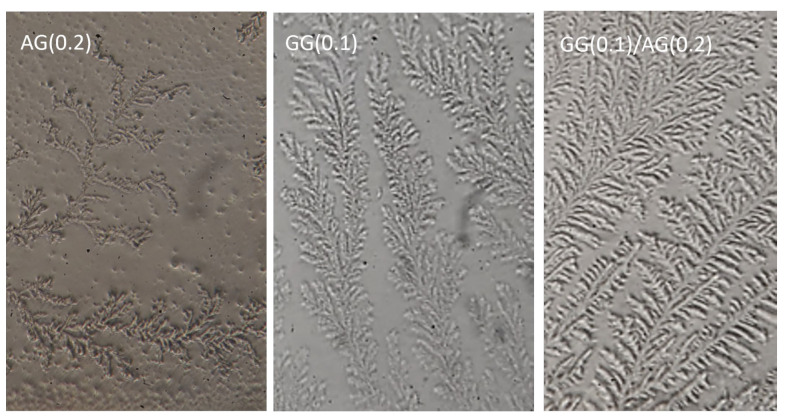
Photomicrographs of ferns formed by the evaporation of 0.2% AG (AG(0.2)) solution, 0.1% GG (GG(0.1)) dispersion, and GG(0.1)/AG(0.2) in situ gelling formulation (40× *g* magnification).

At a concentration of 0.2%, AG was able to form ferns, even if less developed than those observed at higher concentrations. Figure 7 shows the ferning pattern formed by the 0.2% AG aqueous dispersion, which corresponds to type III ferns. These ferns are sparsely branched, with wide spaces between them indicating a less robust interaction between mucins and salts into tears. Similarly, GG was tested alone at a concentration of 0.1% and the ferning pattern obtained also corresponds to type III. The ferns formed by GG are small and poorly developed, with minimal branching and notable empty spaces. However, the combination of AG and GG in the formulation GG(0.1)/AG(0.2) resulted in a more robust fern formation, whose conformation corresponds to type II, characterized by smaller but more branched ferns with reduced spacing between them. This pattern closely resembles the structures formed by natural tear fluid, suggesting that the mixing of AG and GG shows muco-mimetic behavior. Therefore, the formulation developed has characteristics that make it usable as an artificial tear by promoting the stability of the tear film while maintaining its structure.

#### 2.2.2. Mucoadhesion

The ability of GG(0.1)/AG(0.2) to adhere to a mucous surface was evaluated in vitro using a gastric mucin dispersion adsorbed on a filter paper support as a substrate and was expressed as work of adhesion (W) normalized to the contact surface between the formulation and the mucous substrate. As a comparison, the mucoadhesion of an 0.4% HA dispersion was also evaluated. The measured W values for the GG(0.1)/AG(0.2) formulation was 353.17 ± 15.88 erg/cm^2^, showing mucoadhesive capabilities significantly superior (unpaired *t*-test, *p* < 0.5) to those of HA (276.42 ± 14.73 erg/cm^2^), which is known to be a good mucoadhesive ingredient. GG(0.1)/AG(0.2) maintained the same properties even after mixing with ATF producing W values of 368.74 ± 24.92 erg/cm^2^. Ensuring that the formulation obtained by mixing GG and AG maintained its mucoadhesive characteristics was essential although this feature is already known for the two individual components [9,35,36]. Moreover, Burgalassi and colleagues [9] had already demonstrated the superior mucoadhesiveness of AG compared to HA using a rheological interaction method.

Mucoadhesive properties are desirable in formulations intended as artificial tears as this characteristic allows them to remain on the ocular surface longer and therefore perform their hydrating and lubricating activity for a longer time.

#### 2.2.3. Cytotoxicity Assay

Before in vivo evaluation, the toxicity level of the GG(0.1)/AG(0.2) formulation on the rabbit corneal epithelial (RCE) cell monolayer was determined. This method allows us to estimate the number of viable cells present in culture and therefore to evaluate the effect of treatment with a potential toxic agent on the viability of the cell population.

**Figure 8:**
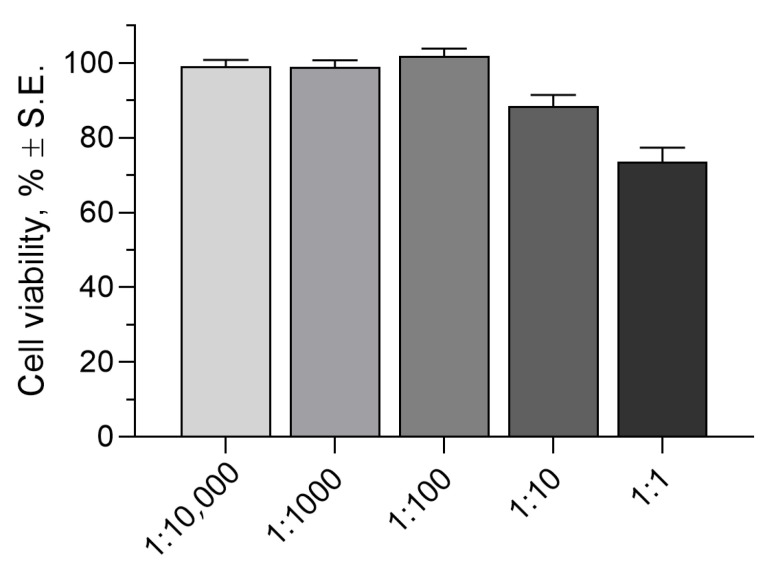
Cell viability of rabbit corneal cells after exposure for 1 h to GG(0.1)/AG(0.2) formulation at different dilution in culture medium.

The assay results are shown in Figure 8 as cell viability after one hour of contact with increasing GG(0.1)/AG(0.2) concentrations in growth medium.

It is noteworthy that all tested concentrations produce cell viability well above 50%, demonstrating the product good biocompatibility with the corneal surface. The highest dilutions all show cell viability values around 100%, comparable to untreated control. From the 1:10 dilution, a decreasing trend is noted, and the 1:1 dilution of the formulation shows the lowest cell viability values (73.61 ± 3.69%) but still indicates good biocompatibility. It should also be noted that the 1:1 dilution reaches gelation conditions due to the presence of an adequate quantity of salts in the growth medium. This factor certainly creates an unfavorable environment for the cells, which are completely covered by a layer of material with reduced oxygen permeability, even if the contact lasts only one hour.

#### 2.2.4. Evaluation of the Time of Residence of the Formulation in the Rabbit Eyes

To evaluate the residence time of the formulation in the precorneal area, AG was derivatized with a fluorescent probe (fluorescein isothiocyanate, FITC). The GG(0.1)/FITC-AG(0.2) formulation containing labeled arabinogalactan instead of the natural product was then prepared; a 0.2% FITC-AG solution in BS1 was used as a reference. Both solutions were sterilized by filtration (membrane pore size 0.2 μm, Minisart, Sartorius SpA, Florence, Italy).

**Figure 9:**
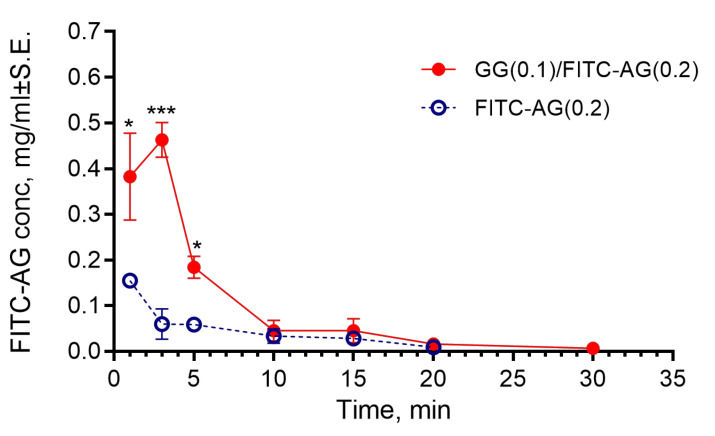
Tear fluid concentration vs. time profiles of fluorescein isothiocyanate-labeled AG after administration of FITC-AG(0.2) solution and GG(0.1)/FITC-AG(0.2) in situ gelling formulation. (*), (***) Significantly different from the same time of reference—*p* < 0.05 and *p* < 0.001, respectively (one-way ANOVA; Tukey’s multiple comparisons test).

The in vivo kinetic profiles are shown in Figure 9. In the FITC-AG(0.2)-treated group, the maximum AG concentration (Cmax = 0.155 ± 0.011 mg/mL) in rabbit tear fluid coincided with the 1 min sampling point and was followed by an exponential decrease until 20 min, the last quantitatively detectable point, reflecting the usual pattern of elimination of a soluble product from the precorneal area.

On the other hand, the in situ gelling formulation reached an FITC-AG concentration of 0.463 ± 0.038 mg/mL within the first 3 min after administration, approximately 3 times higher than the peak concentration reached by the reference and remaining significantly higher than FITC-AG at 5 min. At subsequent time points, the profile overlapped with that of FITC-AG but remained quantifiable up to 30 min.

The total exposure (area under the curve, AUC) for the in situ gelling formulation was 2.56 ± 0.30 mg/mL min^−1^, compared to 0.81 ± 0.11 mg/mL min^−1^ for the FITC-AG(0.2) dispersion, indicating more than a three-fold increase in residence time with GG(0.1)/FITC-AG(0.2). In a paired *t*-test analysis, the mean difference (GG(0.1)/FITC-AG(0.2) minus FITC-AG(0.2)) in AUC was +1.647 (SD of differences = 0.8624), and *p* = 0.0316. The within-subjects effect size, d_z_ = 1.91, demonstrates a very large and biologically significant advantage of the in situ gelling formulation.

The higher FITC-AG concentrations found after ocular administration of the in situ gelling formulation could be the result of a greater initial saturation of the tear film and a slower elimination of the product from the eye, due to the increased viscosity after gelation in the presence of tear fluid salts. Furthermore, it is also plausible that its mucoadhesive properties contribute to the longer permanence in the precorneal area.

It should also be noted that during the in vivo experiment, no hyper lacrimation, ocular redness, eyelid closure, or other signs of irritation or intolerance were observed in the rabbits.

## 3. Conclusions

This study successfully developed an ion-sensitive in situ gelling formulation based on low-acyl gellan gum (GG) and arabinogalactan (AG) to address key limitations of conventional ocular lubricants in the management of Dry Eye Disease (DED). AG has previously demonstrated the ability to promote corneal re-epithelialization and ocular surface repair [9,10]. However, its intrinsic Newtonian flow properties, even at high concentrations, limit its performance in topical ophthalmic delivery, where prolonged surface retention is critical. By leveraging the ion-responsive properties of GG and the bioactivity of AG, the optimized formulation addresses the major limitations of conventional artificial tears, including short ocular residence time and limited bioadhesion [37,38,39]. In fact, these kinds of systems are designed to be easy to apply and to improve the residence time in the eye by forming a three-dimensional gel structure upon contact with tear fluid, thereby significantly increasing the therapeutic efficacy [3]. By fine tuning buffer composition, the optimized formulation GG(0.1)/AG(0.2) achieved rheological characteristics suitable for ocular use. While AG alone exhibits Newtonian behavior, the pseudoplastic behavior is consistent with mechanisms reported in gellan-based gels [37,40]. Response surface analysis highlighted that GG primarily governs viscosity, while AG modulates the elastic properties of the gel network leading to the formation of a robust and stable three-dimensional network which provide superior mucoadhesion and reduced nasolacrimal drainage.

The ferning test revealed that the GG(0.1)/AG(0.2) forms more organized, branched crystalline patterns (Type II), which do not merely indicate compatibility with tear fluid but suggest muco-mimetic behavior. By structurally mimicking tear mucins, the formulation may contribute to restoring the mucin layer in DED patients, thereby supporting tear film stability and improving ocular surface hydration [25,31].

Importantly, thermal (DSC) and particle size analyses (DLS) demonstrate that GG and AG interact structurally in the mixed formulation: the less defined melting peak in the DSC thermogram and the increase in hydrodynamic size upon mixing support the interaction and partial disruption of GG crystalline domains. Furthermore, the formulation remains physically stable under sterilizing filtration.

Cytotoxicity tests on rabbit corneal epithelial cells confirmed that the formulation has good biocompatibility, with cell viability well above critical thresholds, even at the highest concentration, and the in vivo experiment did not reveal any signs of irritation or intolerance, further supporting the safety profile of GG(0.1)/AG(0.2).

Incorporation into a GG-based gel enhances AG bioavailability in the rabbit’s precorneal area but may also reduce dosing frequency, in line with current trends in ocular drug delivery aimed at enhancing both efficacy and patient compliance [41,42,43].

GG(0.1)/AG(0.2) formulation merges the healing properties of AG with the reliable ion-activated gelation of GG. The results support the potential of GG/AG combination as a promising platform for advanced artificial tears or drug loaded ocular formulations that mimic tear-film biomechanics and enhance therapeutic duration of treatment for ocular surface diseases.

Future investigations should focus on the long-term safety, tolerability, and clinical efficacy of the formulation in human subjects and explore its potential to carry active pharmaceutical agents for targeted ocular therapies.

## 4. Materials and Methods

### 4.1. Materials

The materials used in this study included the following: low-acyl gellan gum (GG-LA, Kelcogel^®^, CP Kelco, Atlanta, GA, USA); arabinogalactan extracted from Larix species (AG, kindly provided by Opocrin, Modena, Italy); anhydrous citric acid, ethylenediaminetetraacetic acid (EDTA), anhydrous calcium chloride (CaCl_2_), potassium chloride (KCl), sodium bicarbonate (NaHCO_3_), sodium chloride (NaCl), and mannitol (Carlo Erba Reagents, Milan, Italy); benzalkonium chloride (BAK, Emprove^®^ essential Ph. Eur.), sodium phosphate dibasic heptahydrate (Na_2_HPO_4_·7H_2_O, Emprove^®^ exp.), low-molecular-weight sodium hyaluronate (HA, Ph. Eur. Standard), isothiocyanatofluorescein, and dibutyltin dilaurate (Merck, Darmstadt, Germany); and hog gastric mucin (HGM, Carl Roth GmbH Co. KG, Karlsruhe, Germany). All salts used for the preparation of buffer solutions and artificial tear fluid (ATF) and solvent were of analytical grade. Water was purified using the MilliQ apparatus (Millipore^®^, Milan, Italy).

### 4.2. Methods

#### 4.2.1. Osmolality and pH Measurements

For each prepared formulation, the pH and osmolality were measured. Osmolality was determined using a digital micro-osmometer (Model 5R, Hermann Roebling, Berlin, Germany) based on the freezing point depression method. The pH of the solutions was measured with a digital pH meter (SevenCompact S220, Mettler-Toledo SpA, Milan, Italy). Both sets of measurements were made in triplicate.

#### 4.2.2. Wettability Assessment

The wettability of the formulations was assessed by measuring the contact angles, both before and after dilution with artificial tear fluid (ATF). To simulate the physiological conditions following instillation into the conjunctival sac, the polymeric formulations were mixed with ATF at a ratio of 30:7, mimicking the in vivo dilution of 30 µL of instilled gel with the typical resident tear volume of approximately 7 µL, as described by [20]. The static contact angle, defined as the angle between the solid surface and the tangent at the liquid–air interface where the formulation drop contacts the surface, was measured using an OCA 15 instrument (DataPhysics Instrument, Filderstadt, Germany). The system consisted of a high-resolution CCD video camera and a six-fold power zoom lens with integrated fine focusing; the images were recorded and analyzed by SCA20 software. The measurements were performed using the sessile drop method, in which a known volume of the formulation was deposited on the solid surface (microscope slide) by using a needle with an external diameter of 0.90 mm and an internal diameter of 0.60 mm. For undiluted formulations, a drop volume of 6 µL was used, while for diluted formulations, drops of 9 µL were applied at a flow rate of 0.50 µL/s. When the spreading of the droplet attained an equilibrium state, the contact angle was determined; data are expressed as the mean of the left and right angle on ten replicates.

Wettability was classified based on the contact angle values, ranging from 0° (indicating perfect wettability) to 180° (indicating no wettability); a liquid showing a contact angle less than 90° is understood to wet the surface.

#### 4.2.3. Rheological Analysis

The rheological properties of the polymer solutions were assessed using a rotational rheometer (Rheostress RS150, Haake, NJ, USA) equipped with coaxial cylinders (Z40 and Z41). The rheological behavior of the formulations containing 0.1% GG in the presence of 0.2 and 0.3% AG or otherwise, was investigated before and after dilution with ATF in a ratio of 30:7. Viscosity measurements were performed in triplicate, at 32.0 ± 0.5 °C, with a shear rate (D) ranging from 0 to 200 s^−1^. The viscosity flow curves were recorded and analyzed by using Haake RheoWin™ Software (Version 4.61, Thermo Fisher Scientific, Potsdam, Germany) and the power-law (Ostwald-de-Waele model):

τ = η′ D^N^

where τ is the shear stress, η′ is the apparent viscosity, and N is the flow index.

The viscoelastic properties of the same formulations were investigated through oscillatory measurements, performed in two steps:

- Stress Sweep analysis conducted at 32.0 °C ± 2.0 °C, with shear stress (τ) increasing from 0.1 to 100 Pa, while the frequency was kept at 5 Hz, to determine the linear viscoelastic region (LVR).
- Frequency Sweep analysis conducted at 32.0 ± 2.0 °C, with a frequency range of 0.1–10 Hz and a constant oscillatory stress of 1 Pa to determine the elastic modulus (G′), viscous modulus (G″), and phase angle (δ) as a function of the frequency.

#### 4.2.4. Dynamic Light Scattering Analysis

The intensity-weighted mean hydrodynamic size (Z-Ave) and polydispersity index (PdI) of the samples were determined by Dynamic Light Scattering (DLS) (Zetasizer Pro, Malvern Panalytical, Malvern, UK) after a suitable dilution of the polymer dispersions with freshly collected ultrapure water (Milli-Q Plus, Millipore, Milan, Italy) to ensure that their concentration fell within the optimal measurement intensity range. Measurements were conducted at 25 °C after an equilibration time of 120 s, with three runs for each sample, using a backscatter angle of 173°.

#### 4.2.5. Preparation of Artificial Tear Fluid (ATF)

The artificial tear fluid (ATF) with a pH = 7.46 was prepared following the composition reported by Kotreka and colleagues [20]: 6.800 g/L sodium chloride (NaCl), 2.200 g/L sodium bicarbonate (NaHCO_3_), 1.400 g/L potassium chloride (KCl), and 0.084 g/L calcium chloride dihydrate (CaCl_2_·2H_2_O). The salts were accurately weighed and dissolved in purified water in a volumetric flask, and the solution was diluted to the final volume. The pH was adjusted to 7.46 by adding a few drops of 1N hydrochloric acid. The prepared lacrimal fluid was stored at room temperature.

#### 4.2.6. Design of Experiments (DOE): Optimization Study for the Selection of the Most Performant In Situ Gelling Formulation

To establish the Design of Experiment (DoE) framework, formulations were prepared with GG at concentrations of 0.05%, 0.10%, and 0.20% *w*/*w*, as well as combinations of GG with AG at 0.20% and 0.30% *w*/*w*. These formulations were then evaluated for viscosity and viscoelasticity, with a focus on the elastic modulus (G′) and viscous modulus (G″). A 3 × 3 full-factorial design was planned, incorporating two factors at three levels each (Table 7).

The independent variables (factors) were the concentrations of AG (X_1_) and GG (X_2_), while the dependent variables were viscosity at a shear rate of 2.5 s^−1^, along with the viscoelastic properties (G′ and G″) calculated at 1 Hz. This DoE approach enabled a systematic investigation and optimization of the formulations, providing detailed insights into how varying concentrations of AG and GG affected their rheological behavior.

#### 4.2.7. Thermal Behavior

Thermal analysis was conducted on the DoE-selected formulation—GG(0.1)/AG(0.2)—and on the single polymer dispersions using differential scanning calorimetry (DSC). This method provided insights into the thermal Behavior and stability of polymer formulations under varying thermal conditions. The DSC measurements were performed with a PerkinElmer DSC 4000 (Perkin Elmer, Milan, Italy). A sample weighing approximately 2–4 mg was carefully placed in a flat-bottomed aluminum pan and heated at a constant rate of 10 °C/min in a range from 5 °C to 85 °C under a nitrogen purge gas flow of 20 mL/min. The thermal profiles were recorded with Pyris Instrument Managing Software (Version 3.8. Perkin Elmer, Milan, Italy), and the analysis was performed using OriginPro^®^ software (Version 2018, OriginLab Co., Northampton, MA, USA).

#### 4.2.8. Ferning Test

The ferning test was performed on formulations containing both 0.2% AG and 0.1% GG and a combination of them after always mixing with ATF. For each test, 10 µL of the formulation were mixed with 2 µL of artificial tear fluid on a microscope slide. The samples were left to dry at room temperature (25 °C ± 1 °C) for 24 h and photographed under an optical microscope (Reichert-Jung MicroStar 120; Leica Biosystems Nussloch GmbH, Baden-Württemberg, Germany) at 40× *g* magnification.

#### 4.2.9. Mucoadhesion Test

The mucoadhesive properties of GG(0.1)/AG(0.2) before and after dilution with ATF were evaluated by measuring their work of adhesion (W) on a mucous surface by tensile test. The apparatus consisted of a testing cell (two cylindrical sections, upper and lower) connected to a tensile apparatus fitted with force and elongation transducers, whose output was fed to a computer equipped with data acquisition software (Handyscope2, TiePie Engineering, Sneek, The Netherlands) [22]. The mucous layer consisted of 0.125 mL of a 28.0% *w*/*w* aqueous dispersion of HGM uniformly spread on wet filter paper disks of 12 mm diameter tightly secured to both cell sections. Following application, the mucin layers were superficially dried for 5 min by cold air, and then 50 µL of the semisolid sample under study was thinly layered onto upper mucous surface. The lower cell section was slowly raised and put into contact with the sample; after 1 min of contact, the cell sections were moved away at constant speed (1.25 mm/min) up to complete separation. Analysis of the resulting force versus distance curves (work of adhesion, W) was performed using Prism^®^ software (GraphPad Software Inc., La Jolla, CA, USA). All W values were normalized with respect to the adhesion area; twelve repetitions were made for each polymer.

#### 4.2.10. Cytotoxicity Assay

A cytotoxicity test was carried out on rabbit corneal epithelial cells (RCE) using the ready-to-use cell proliferation reagent WST-1. The RCE cells were plated at 3 × 10^5^ cells/well, in 96-well microtiter plates. After 24 h, the medium was completely aspirated; 100 μL of GG(0.1)/AG(0.2) formulation suitably diluted with growth medium was added to each well and maintained in contact with the cells for 60 min. Subsequently, the formulation was removed, and the cells were washed three times with DMEM/F12. Then, 100 μL of fresh growth medium and 10 μL of reagent WST-1 were added in each well, the cells were incubated for 2 h at 37 °C in a humidified atmosphere with 5% CO_2_, the microplate was thoroughly shaken for 1 min, and finally, the absorbance was determined at 450 nm using a microtiter reader (Asys UVM 340; Biochrom, Cambridge, UK).

The background absorbance was measured on wells containing only the growth medium and WST-1, and the results were expressed as a percentage of the absorbance of treated versus no-treated wells (control, 100%).

#### 4.2.11. Evaluation of the Time of Residence of the Formulation in the Rabbit Eyes

##### Fluorescein Isothiocyanate AG Synthesis

Fluorescein isothiocyanate-labeled AG (FITC-AG) was synthesized as follows: 1.0 g of AG was dissolved in methylsulfoxide (10 mL) containing a few drops of pyridine. Fluorescein isothiocyanate (0.1 g) and dibutylin dilaurate (20 mg) were added, and the mixture was heated at 95 °C for 2 h. This is followed by several precipitations with ethanol to remove free dye before filtering off FITC-AG and drying it at 80 °C. FITC-AG solution was obtained by heating at 80 °C for 30 min under gently stirring.

##### Animal Testing

Six albino New Zealand rabbits, weighing 3.0–3.5 kg (Pamaploni rabbitry, Fauglia, Italy), were treated in according to “Guide for the Care and Use of Laboratory Animals”. For all the experimental procedures, the ARVO Statement for the Use of Animals in Ophthalmic and Vision Research and the European Union guidelines for the use of animals in research were applied. Furthermore, the experimental protocol was approved by the Ethical and Scientific Committee of the University of Pisa and by the Italian Minister of Health. The rabbits were housed in standard cages in a light-controlled room at 19 ± 1 °C and 50 ± 5% R.H. with standard pellet diet and water ad libitum. During the experiment, the animals were placed in restraining boxes in a room with dim lighting; they could move their heads freely, and eye movements were not restricted.

A total of 50 μL of 0.2% FITC-AG solution and its combination with 0.1% GG (GG(0.1)/FITC-AG(0.2)) were administered into the lower conjunctival sac of one eye of each of the 6 rabbits (three animals per formulation, with a repeated administration after one week, ensuring crossover between animals), and their eyelids were gently kept closed for 30 sec. At determined time intervals (1, 3, 5, 10, 15, 20, 30 min) after administration, tear fluid samples were collected from the lower marginal tear strip using 1.0 μL disposable glass capillaries (Drummond Microcaps, Fisher Scientific, St. Louis, MO, USA). The tear fluid was transferred into microtubes, and the capillaries were flushed with distilled water. Then, the samples were appropriately diluted and analyzed to quantify FITC-AG using a Varioskan multimode microplate reader (Thermo Fisher Scientific Inc., Segrate, Italy) at λem = 514 nm and λex = 490 nm.

#### 4.2.12. Statistical Analysis

Statistical analyses for multiple groups were determined by a one-way ANOVA test and Tukey’s test for multiple comparisons. Statistical significance was defined as follows: * *p* < 0.05, ** *p* < 0.01, *** *p* < 0.001, **** *p* < 0.0001.

Rheological data (viscosity, elastic modulus G′, viscous modulus G″) were analyzed by polynomial response-surface regression. The model included linear, quadratic, and interaction terms for concentrations of the two polymers (GG and AG). Goodness-of-fit was assessed via a coefficient of determination (R^2^) and ANOVA, and multicollinearity among predictors was evaluated using variance inflation factors (VIF).

All processing was performed using GraphPad Prism Software, version 10 (GraphPad, San Diego, CA, USA).

## Figures and Tables

**Table 1:** Composition of different buffers tested and ions concentrations.

Buffer	Na_2_HPO_4_·7H_2_O (mM)	Citric Acid (mM)	NaCl (mM)	CaCl_2_ (mM)	KCl (mM)	Mannitol (mM)	Monovalent Cations (mM)	Ca^2+^ (mM)	Total Cations (mM)
BS1	10.00	0.73	1.71	0.76	18.78	217.38	40.49	0.76	41.25
BS2	10.00	0.73	1.71	2.00	18.78	217.38	40.49	2.00	42.49
BS3	10.00	0.73	1.71	-	18.78	217.38	40.49	-	40.49

**Table 2:** Characteristics of the formulations prepared with different buffer solutions in terms of pH, osmolality, and viscosity (mean ± SE; *n* = 3).

Formulations	Cations (mM)	Mannitol (mM)	pH	Osmolality (mOsmol/kg)	Viscosity (mPa·s)	
					Before ATF Dilution	After ATF Dilution
GG(0.1)/BS1	41.25	217.38	6.45 ± 0.02	299 ± 0.58	28.62 ± 0.66 ^####^	31.27 ± 0.64 *
GG(0.1/BS2	42.49	217.38	6.97 ± 0.01	297 ± 0.58	28.54 ± 2.11 ^###^	26.48± 1.09
GG(0.1)/BS3	40.49	217.38	6.73 ± 0.01	302 ± 1.00	2.18 ± 1.02	8.97 ± 0.99 **

(*), (**) Significantly different from the same formulation without ATF; *p* < 0.05 and *p* < 0.01, respectively. (^###^), (^####^) Significantly different from GG(0.1)/BS3 formulation before ATF dilution; *p* < 0.001 and *p* < 0.0001, respectively (one-way ANOVA and Tukey’s multiple comparisons test).

**Table 3:** Composition of the GG- and AG-based formulations under study.

Formulation	GG (% *w*/*w*)	AG (% *w*/*w*)	Na_2_HPO_4_· 7H_2_O (mM)	Citric Acid (mM)	NaCl (mM)	CaCl_2_ (mM)	KCl (mM)	Mannitol (mM)	EDTA (% *w*/*w*)	BAK (% *w*/*w*)
GG(0.05)	0.05	-	10.00	0.73	1.71	0.76	18.78	217.38	0.05	0.005
GG(0.05)/AG(0.2)	0.05	0.2	10.00	0.73	1.71	0.76	18.78	217.38	0.05	0.005
GG(0.05)/AG(0.3)	0.05	0.3	10.00	0.73	1.71	0.76	18.78	217.38	0.05	0.005
GG(0.1)	0.1	-	10.00	0.73	1.71	0.76	18.78	217.38	0.05	0.005
GG(0.1)/AG(0.2)	0.1	0.2	10.00	0.73	1.71	0.76	18.78	217.38	0.05	0.005
GG(0.1)/AG(0.3)	0.1	0.3	10.00	0.73	1.71	0.76	18.78	217.38	0.05	0.005
GG(0.2)	0.2	-	10.00	0.73	1.71	0.76	18.78	217.38	0.05	0.005
GG(0.2)/AG(0.2)	0.2	0.2	10.00	0.73	1.71	0.76	18.78	217.38	0.05	0.005
GG(0.2)/AG(0.3)	0.2	0.3	10.00	0.73	1.71	0.76	18.78	217.38	0.05	0.005

**Table 4:** Characterization parameters of the GG- and AG-based formulations under study.

Formulation	pH (±SE)	Osmolality (mOsmol/kg ±SE)	Wettability (θ, ° ±SE)		Viscosity (mPa. s ±SE)		Increase Factor (IF)	IF Mean
			Before	After	Before	After		
			ATF Dilution	ATF Dilution	ATF Dilution	ATF Dilution		
GG(0.05)	6.18 ± 0.03	302.0 ± 1.00	56.10 ± 2.96	54.50 ± 1.01	9.05 ± 1.12	14.27 ± 0.83	1.58	
GG(0.05)/AG(0.2)	6.12 ± 0.02	297.3 ± 1.20	57.60 ± 1.64	59.10 ± 2.32	8.12 ± 0.86	16.52 ± 1.59	2.03	
GG(0.05)/AG(0.3)	6.28 **±** 0.05	299.0 ± 0.58	54.90 ± 3.10	56.20 ± 3.00	12.04 ± 0.35	25.87 ± 1.31	2.15	
								1.92
GG(0.1)	6.42 ± 0.03	291.0 ± 1.00	49.90 ± 1.61	54.70 ± 1.74	14.07 ± 0.43	34.82 ± 1.02	2.47	
GG(0.1)/AG(0.2)	6.08 ± 0.02	301.3 ± 0.88	52.50 ± 1.12	51.90 ± 2.35	17.83 ± 1.96	41.66 ± 8.12	2.34	
GG(0.1)/AG(0.3)	5.96 ± 0.06	305.7 ± 0.67	51.30 ± 1.13	49.60 ± 1.38	19.30 ± 2.96	49.22 ± 2.59	2.55	
								2.45
GG(0.2)	6.12 ± 0.04	300.0 ± 0.58	51.70 ± 3.06	53.20 ± 1.46	106.58 ± 4.35	382.58 ± 6.28	3.59	
GG(0.2)/AG(0.2)	6.20 ± 0.01	298.0 ± 1.00	48.50 ± 1.94	49.40 ± 5.06	95.30 ± 3.86	302.52 ± 2.76	3.17	
GG(0.2)/AG(0.3)	6.32 ± 0.00	309.3 ± 0.67	51.70 ± 1.50	56.50 ± 1.90	105.41 ± 1.39	333.03 ± 4.21	3.16	
								3.31

**Table 5:** Experimental design for the DoE optimization study.

Formulation	Independent Variables Levels		Dependent Variables Values— Before ATF Dilution			Dependent Variables Values— After ATF Dilution		
	X_1_ (AG)	X_2_ (GG)	Viscosity (mPa·s)	Elastic Modulus (G′, Pa)	Viscous Modulus (G″, Pa)	Viscosity (mPa·s)	Elastic Modulus (G′, Pa)	Viscous Modulus (G″, Pa)
GG(0.05)	−1	−1	9.05	0.144	0.042	14.27	0.129	0.047
GG(0.05)/AG(0.2)	0	−1	8.12	0.127	0.042	16.52	0.121	0.061
GG(0.05)/AG(0.3)	+1	−1	12.04	0.125	0.052	25.87	0.121	0.069
GG(0.1)	−1	0	14.07	0.137	0.101	34.82	0.105	0.101
GG(0.1)/AG(0.2)	0	0	17.83	0.141	0.106	41.66	0.092	0.106
GG(0.1)/AG(0.3)	+1	0	19.30	0.136	0.107	49.22	0.088	0.168
GG(0.2)	−1	+1	106.58	0.067	0.258	382.58	0.167	0.300
GG(0.2)/AG(0.2)	0	+1	95.30	0.039	0.273	302.52	0.025	0.298
GG(0.2)/AG(0.3)	+1	+1	105.41	0.027	0.268	333.03	0.032	0.384

**Table 6:** Physicochemical characteristics of samples under study: size and PDI obtained by dynamic light scattering analysis (mean ± SE: *n* = 3); viscosity (mean ± SE: *n* = 3).

Formulation	Size (nm)	Polydispersity Index	Viscosity (mPa·s)
GG(0.1)	147.3 ± 7.48 ****	0.31	14.07 ± 0.43
AG(0.2)	112.1 ± 3.59 ****	0.28	1.04 ± 0.054
GG(0.1)/AG(0.2)	331.9 ± 16.62	0.34	17.83 ± 1.96
GG(0.1)/AG(0.2) after filtration	295.3 ± 4.27	0.19	20.73 ± 1.71
GG(0.1)/AG(0.2) after 9 months	324.0 ± 16.30	0.22	19.57 ± 0.41

(****) Significantly different from the GG(0.1)/AG(0.2) formulation, even after filtration and storage (one-way ANOVA; Tukey’s multiple comparisons test: *p* < 0.0001).

**Table 7:** Levels for the two independent variables used in the DoE optimization study.

Independent Variables	Levels		
	+1	0	−1
X_1_ = AG % *w*/*w*	0.3	0.2	0
X_2_ = GG % *w*/*w*	0.2	0.1	0.05

## Data Availability

The raw data are available from the corresponding author upon reasonable request.
